# Supplementary material for: An orally bioavailable broad-spectrum antiviral inhibits SARS-CoV-2 in human airway epithelial cell cultures and multiple coronaviruses in mice
Source: Sci Transl Med. 2020 Apr 6:eabb5883. doi: 10.1126/scitranslmed.abb5883 (PMC7164393; doi:10.1126/scitranslmed.abb5883)
Supplement: Supplementary file 1 [file abb5883-SM.pdf]

Science Translational Medicine
AAAS

stm.sciencemag.org/cgi/content/full/scitranslmed.abb5883/DC1

Supplementary Materials for

An orally bioavailable broad-spectrum antiviral inhibits SARS-CoV-2 in human airway epithelial cell cultures and multiple coronaviruses in mice

Timothy P. Sheahan*, Amy C. Sims, Shuntai Zhou, Rachel L. Graham, Andrea J. Pruijssers, Maria L. Agostini, Sarah R. Leist, Alexandra Schäfer, Kenneth H. Dinnon III, Laura J. Stevens, James D. Chappell, Xiaotao Lu, Tia M. Hughes, Amelia S. George, Collin S. Hill, Stephanie A. Montgomery, Ariane J. Brown, Gregory R. Bluemling, Michael G. Natchus, Manohar Saindane, Alexander A. Kolykhalov, George Painter, Jennifer Harcourt, Azaibi Tamin, Natalie J. Thornburg, Ronald Swanstrom, Mark R. Denison, Ralph S. Baric*

*Corresponding authors: sheahan@email.unc.edu and rbaric@email.unc.edu

Published 6 April 2020 on *Science Translational Medicine* First Release
DOI: 10.1126/scitranslmed.abb5883

This PDF file includes:

Supplementary Figure 1. Assessment of cytotoxicity of NHC in primary human epithelial cell cultures by qRT-PCR.
Supplementary Figure 2. High conservation of RdRp functional domains for SARS-CoV-2.
Supplementary Figure 3. Prophylactic EIDD-2801 reduces SARS-CoV replication and pathogenesis.
Supplementary Figure 4. Prophylactic EIDD-2801 reduces MERS-CoV replication and pathogenesis.
Supplementary Table 1. Real-time PCR primer/probe sets for indicators of cellular apoptosis/toxicity.
Supplementary Table 2. Primers used for MiSeq library prep and sequencing.

Other Supplementary Material for this manuscript includes the following:
(available at stm.sciencemag.org/cgi/content/full/scitranslmed.abb5883/DC1)

Data file S1. Primary data.

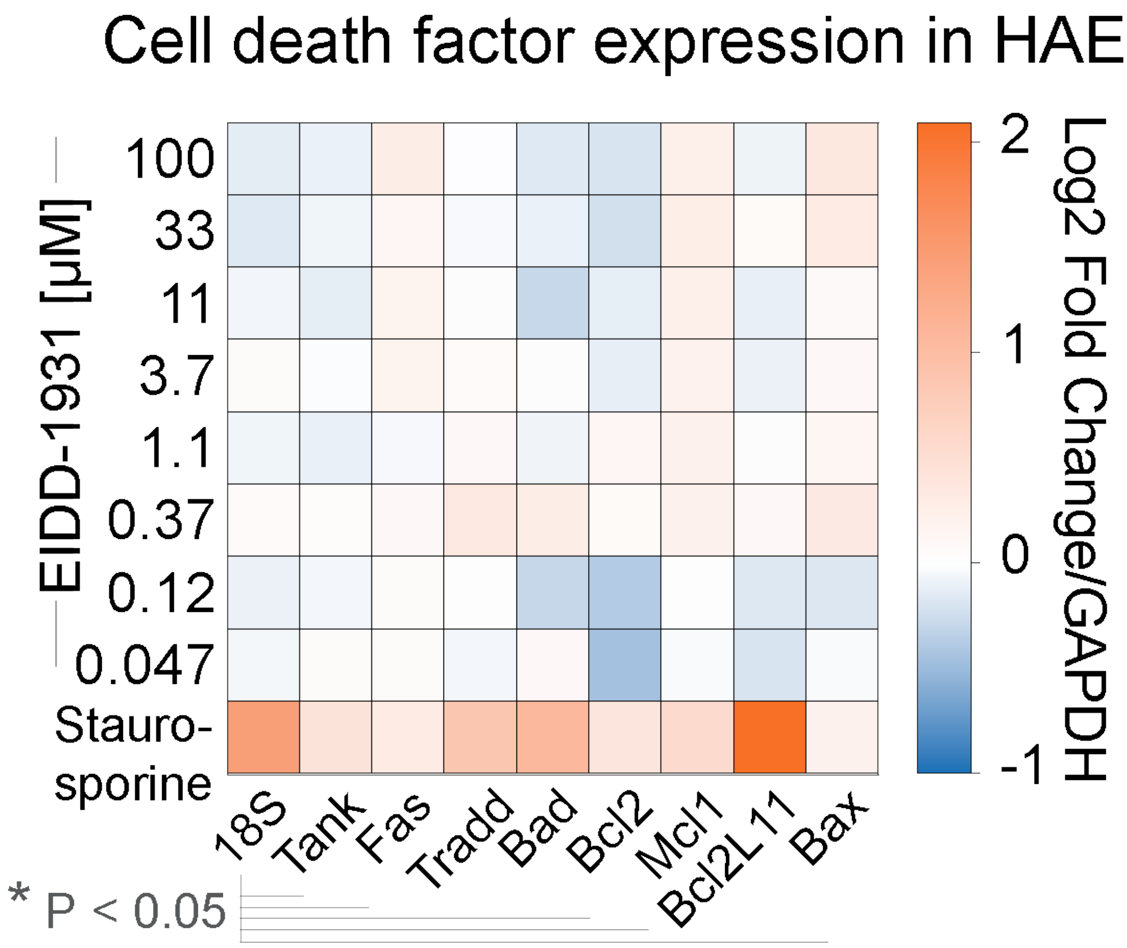

Supplementary Figure 1: Assessment of cytotoxicity of NHC in primary human epithelial cell cultures by qRT-PCR. Companion figure to **Figure 2**. Primary human epithelial cell cultures were exposed to positive control 1µM staurosporine or a dose response of NHC for 48 hr. Cytotoxicity was assessed by qRT-PCR for cell death factor gene expression. Studies were performed in two different human donors. Data from one donor is shown. Differences were determined by two-way ANOVA with a Dunnett's multiple comparison test.

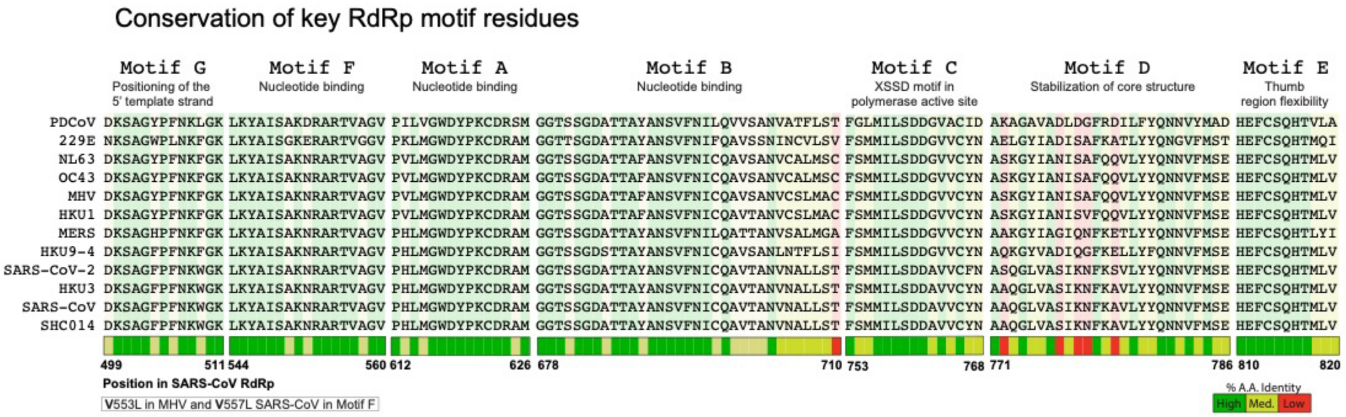

Supplementary Figure 2: High conservation of RdRp functional domains for SARS-CoV-2. Companion figure to **Figure 3.** Multiple sequence alignment of the RNA dependent RNA polymerase (RdRp) from viruses in the dendrogram in Fig. 3A.

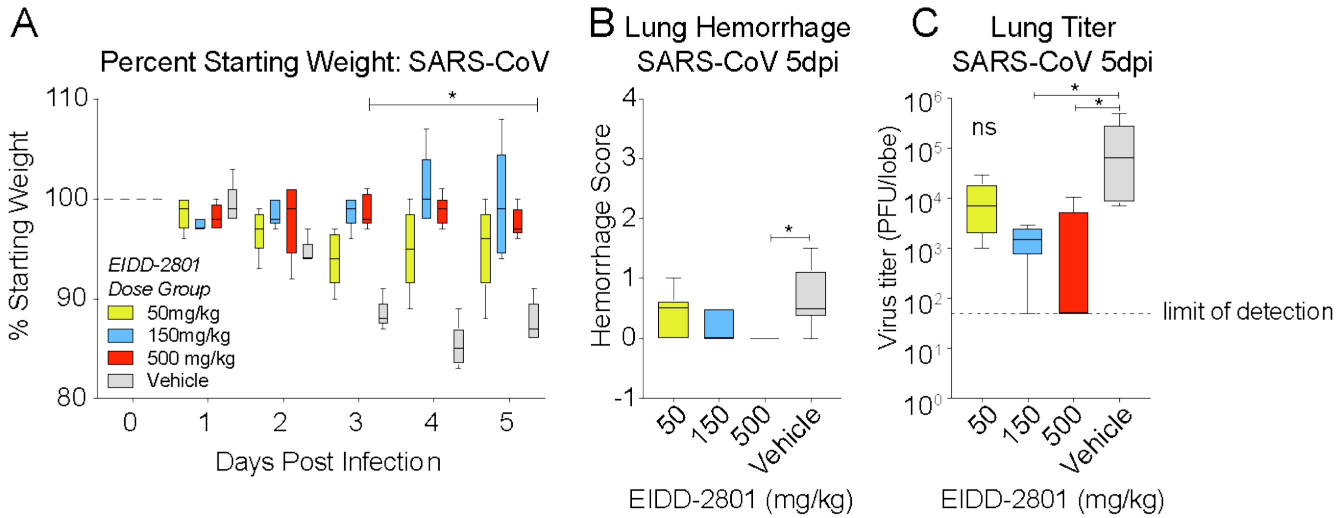

Supplementary Figure 3: Prophylactic EIDD-2801 reduces SARS-CoV replication and pathogenesis. Companion figure to **Figure 6**. Equivalent numbers of 20 week old male and female C57BL/6 mice were administered vehicle (10% PEG, 2.5% Cremophor RH40 in water) or NHC prodrug EIDD-2801 beginning at 2 hr prior to infection and every 12 hr thereafter by oral gavage (n = 10/group). Mice were intranasally infected with 1E+04 PFU mouse-adapted SARS-CoV MA15 strain. **(A)** Percent starting weight. Asterisks indicate differences from vehicle by two-way ANOVA with Dunnett's multiple comparison test. **(B)** Lung hemorrhage in mice from panel **A** scored on a scale of 0-4 where 0 is a normal pink healthy lung and 4 is a diffusely discolored dark red lung. **(C)** Virus lung titer in mice from panel **A** as determined by plaque assay. Asterisks in both panel **B** and **C** indicate differences by Kruskal-Wallis with a Dunn's multiple comparison test: *, significantly different from vehicle.

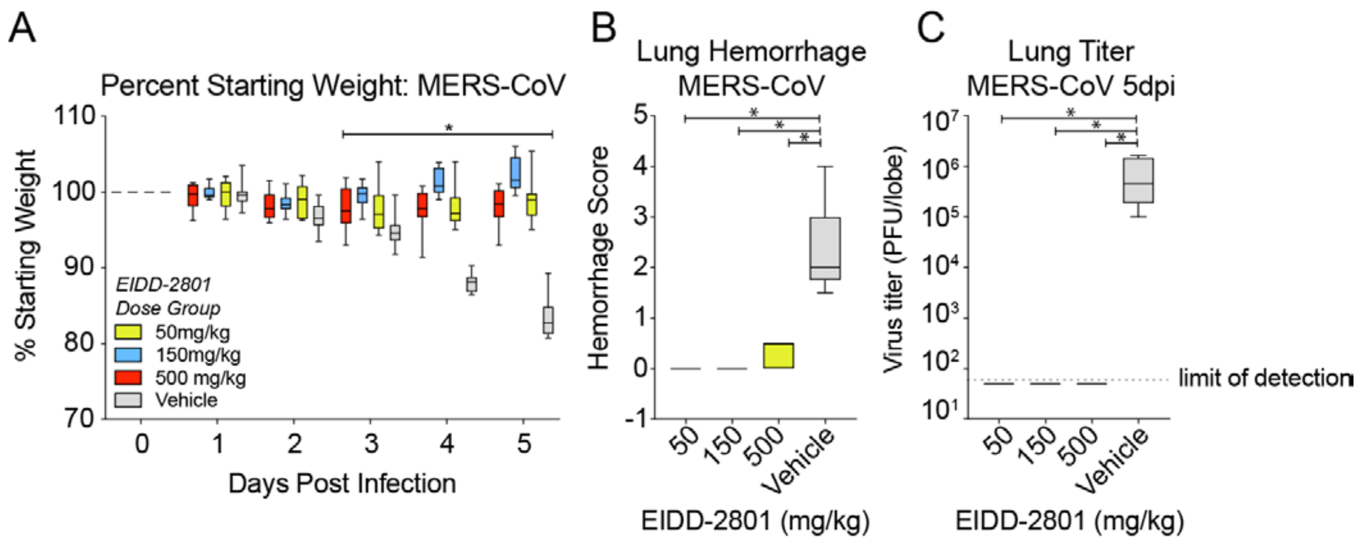

Supplementary Figure 4: Prophylactic EIDD-2801 reduces MERS-CoV replication and pathogenesis. Companion figure to **Figure 7.** Equivalent numbers of 10-14 week old male and female C57BL/6 hDPP4 mice were administered vehicle (10% PEG, 2.5% Cremophor RH40 in water) or NHC prodrug EIDD-2801 beginning 2 hr prior to infection every 12 hr thereafter by oral gavage (n = 10/group). Mice were intranasally infected with 5E+04 PFU mouse-adapted MERS-CoV M35C4 strain. **(A)** Percent starting weight. Asterisks indicate differences by two-way ANOVA with Dunnett's multiple comparison test. **(B)** Lung hemorrhage in mice from panel **A** scored on a scale of 0-4 where 0 is a normal pink healthy lung and 4 is a diffusely discolored dark red lung. **(C)** Virus lung titer in mice from panel **A** as determined by plaque assay. Asterisks in both panel **B** and **C** indicate differences by Kruskal-Wallis with Dunn's multiple comparison test: *, significantly different from vehicle.

.

Supplementary Table 1. Real-time PCR primer/probe sets for indicators of cellular apoptosis/toxicity

Primer/Probe Target	Assay Reference Number*
Bax	Hs00180269_m1
Bad	Hs00188930_m1
Bcl2L11	Hs00708019_s1
Bcl2	Hs00608023_m1
Mcl1	Hs01050896_m1
Tradd	Hs00601065_g1
Fas	Hs00236330_m1
Tank	Hs00370305_m1
18S	4352930E
GAPD**	4352934E

* Validated assays available from Life Technologies

** The housekeeping gene hGAPDH was used for normalization of real-time results.

Supplementary Table 2. Primers used for MiSeq library prep and sequencing.

Primer	5'-3'	Comment
41R_PID11	GTGACTGGAGTTCAGACGTGTGCTCTTCCGATCTNNNNNNNNNNNCA GTATGACCTTCCTGTTGCTTCT	cDNA primer. Targeting 20331-20350 on the reference genome.
nsp10_PID11	GTGACTGGAGTTCAGACGTGTGCTCTTCCGATCTNNNNNNNNNNNCA GTCCTAAAGACGACATCAGTGG	cDNA primer. Targeting 13488-13507 on the reference genome.
nsp12_PID11	GTGACTGGAGTTCAGACGTGTGCTCTTCCGATCTNNNNNNNNNNNCA GTATAGCCAAAGACACAAACCG	cDNA primer. Targeting 15983-16002 on the reference genome.
nsp14_PID11	GTGACTGGAGTTCAGACGTGTGCTCTTCCGATCTNNNNNNNNNNNCA GTGAACATCGACAAAGAAAGGG	cDNA primer. Targeting 18715-18734 on the reference genome.
ifit3_PID11	GTGACTGGAGTTCAGACGTGTGCTCTTCCGATCTNNNNNNNNNNNCA GTTTCAGCCACTCCTTTATCCC	cDNA primer. Targeting mice IFIT3 mRNA.
isg15_PID11	GTGACTGGAGTTCAGACGTGTGCTCTTCCGATCTNNNNNNNNNNNCA GTGGGGCTTTAGGCCATACTC	cDNA primer. Targeting mice ISG15 mRNA.
41F_AD	GCCTCCCTCGCGCCATCAGAGATGTGTATAAGAGACAGNNNNGCTAC AAGTTCGTCCTTTGG	1st round PCR forward primer. Targeting 19812-19831 on the reference genome
nsp10_AD	GCCTCCCTCGCGCCATCAGAGATGTGTATAAGAGACAGNNNNTGCTC AGGTGCTAAGCGAAT	1st round PCR forward primer. Targeting 12983-13002 on the reference genome
nsp12_AD	GCCTCCCTCGCGCCATCAGAGATGTGTATAAGAGACAGNNNNATAG GCTTCGATGTTGAGGG	1st round PCR forward primer. Targeting 15388-15407 on the reference genome
nsp14_AD	GCCTCCCTCGCGCCATCAGAGATGTGTATAAGAGACAGNNNNATTGC AAGCTGGTTCTAACA	1st round PCR forward primer. Targeting 18260-18279 on the reference genome
ifit3_AD	GCCTCCCTCGCGCCATCAGAGATGTGTATAAGAGACAGNNNNCGATC CACAGTGAACAACAG	1st round PCR forward primer. Targeting mice IFIT3 mRNA.
isg15_AD	GCCTCCCTCGCGCCATCAGAGATGTGTATAAGAGACAGNNNNTGGG ACCTAAAGGTGAAGATG	1st round PCR forward primer. Targeting mice ISG15 mRNA.
Adapter R	GTGACTGGAGTTCAGACGTGTGCTC	1st round PCR reverse primer
Universal Adapter	AATGATACGGCGACCACCGAGATCTACACGCCTCCCTCGCGCCATCA GAGATGTG	2nd round PCR forward primer with Illumina adapter sequence
Indexed Adapter	CAAGCAGAAGACGGCATACGAGATNNNNNNGTGACTGGAGTTCAGA CGTGTGCTC	2nd round PCR reverse primer with Illumina adapter sequence and indices (NNNNNNN)
Old Nextera	GCCTCCCTCGCGCCATCAGAGATGTGTATAAGAGACAG	Customized sequencing primer
